# Evaluation of Ovarian Function Suppression Failure in Premenopausal Women with Early-Stage Breast Cancer

**DOI:** 10.3390/cancers18081296

**Published:** 2026-04-20

**Authors:** Catherine Côté, Maïka Wild, Lauriann Roussel, Julia Hoang, David Simonyan, Christian Laflamme, Julie Lemieux

**Affiliations:** 1 Department of Medicine, University Laval, Quebec, QC G1V 0A6, Canada; 2 Clinical and Evaluative Research Platform, CHU de Québec-Université Laval Research Centre, Quebec, QC G1V 4G2, Canada; 3 Oncology Axis, CHU de Québec-Université Laval Research Centre, Quebec, QC G1S 4L8, Canada

**Keywords:** gonadotropin-releasing hormone agonist, hormone receptor-positive early breast cancer, ovarian suppression, adjuvant endocrine treatment, premenopausal

## Abstract

**Background/Objectives**: Ovarian function suppression (OFS) is part of endocrine therapy treatment for high-risk premenopausal women with hormone receptor-positive (HR+) breast cancer (BC). Incomplete OFS may occur and compromise treatment efficacy. **Methods**: We conducted a retrospective single-center cohort study of premenopausal patients with HR + BC treated with OFS therapy at the Centre des maladies du sein (CMS) of the CHU de Québec (Québec, Canada). Ovarian function suppression failure was defined as either biochemical failure (estradiol (E2) levels within the premenopausal range according to local immunoassays used) or clinical failure (return of menstrual bleeding). Patients’ characteristics, treatment specifics and side effects, timing and type of OFS failure, recurrence, and mortality were analyzed. **Results**: Among 208 included patients, 17 (8.2%) experienced at least one episode of OFS failure during a median follow-up of 62.6 months. Most failures occurred early, with 76.2% occurring within the first year of treatment. No significant differences were observed between patients with and without OFS failure regarding age, body mass index (BMI), or prior chemotherapy exposure. Patients with OFS failure had a significantly younger age at first pregnancy and higher rates of active smoking. No BC recurrence or death occurred among patients with OFS failure. Treatment-related side effects were common, and 23.0% of OFS regimens were discontinued due to adverse effects. **Conclusions**: In this study, OFS failure occurred in fewer than 10% of premenopausal patients. Younger age at first pregnancy and active smoking may be associated with OFS failure, but further data are needed to validate these exploratory associations. These findings reinforce the need for larger prospective studies to better assess OFS failure and develop standardized monitoring strategies to optimize treatment efficacy.

## Simple Summary

Ovarian function suppression (OFS) is a key component of endocrine treatment for premenopausal patients with hormone receptor-positive breast cancer. Although large clinical trials such as SOFT and TEXT have demonstrated its effectiveness, important questions remain regarding how often ovarian suppression fails, which patients may be at higher risk, and the clinical implications of such failures. In this retrospective cohort study conducted at a single oncology center in Québec, we evaluated 208 premenopausal patients treated with OFS. During a median follow-up of 62.6 months, 17 (8.2%) patients experienced failure, most of which occurred within the first year of treatment. OFS failure was associated with a younger age at first pregnancy and higher rates of active smoking. Overall, these findings suggest that OFS failure occurs and is clinically relevant, highlighting the need for larger prospective studies to better define its determinants and consequences.

## 1. Introduction

Breast cancer (BC) represents the second leading cause of cancer-related death among Canadian women and was the most diagnosed cancer in 2025 [1]. Recent studies indicate that its incidence is rising, particularly among women under 50, who are usually premenopausal [2,3]. Breast cancer is classified by hormone receptor (HR) expression, including estrogen and progesterone receptors, and by human epidermal growth factor receptor 2 (HER2) status [4]. In a cohort study including more than 17,000 women, 62% of those aged 40 years or younger had HR + BC [5].

Several adjuvant endocrine therapies are available for HR + BC [6]. Tamoxifen acts as an estrogen receptor blocker, whereas aromatase inhibitors (AIs) inhibit the peripheral conversion of steroid precursors into estrogen. Gonadotropin-releasing hormone agonists (GnRHas), such as goserelin injections, suppress the release of follicle-stimulating hormone (FSH) and luteinizing hormone (LH), thereby preventing ovarian estrogen production, a mechanism referred to as ovarian function suppression (OFS) [6,7]. SOFT-TEXT, which includes two large randomized controlled clinical trials, demonstrated a significant improvement in disease-free survival when GnRHas were combined with tamoxifen, compared to tamoxifen alone (a difference of 4.3%, *p* = 0.009), as well as an improvement in overall survival (a difference of 1.8%, *p* = 0.01) [8]. Among women who received OFS, the combination of OFS plus exemestane was associated with a 4.6% absolute improvement in 12-year disease-free survival (DFS) compared with OFS plus tamoxifen (*p* < 0.001) [9]. A systematic review including 11,538 premenopausal women with HR + BC also reported that the addition of GnRHas reduced mortality (HR 0.86, 95% CI 0.78 to 0.94) and improved DFS (HR 0.83, 95% CI 0.77 to 0.90) [6].

Despite their proven efficacy, GnRHas are associated with side effects that can meaningfully affect quality of life, such as hot flashes and sexual symptoms, and do not consistently achieve complete OFS in all patients [10,11]. In the SOFT-EST study, 17% of patients experienced OFS failure up to one year after initiating treatment [8]. Incomplete OFS is defined by estradiol (E2) levels within the premenopausal range or by clinical signs of ovarian function recovery, such as the return of menstrual bleeding [12]. However, the prevalence of OFS failure is not well characterized, and its implications for survival or recurrence risk remain uncertain [13]. Large-scale studies providing robust clinical evidence are lacking, and smaller-scale studies are limited in the literature [14,15,16]. A few small retrospective studies have explored factors associated with a higher risk of failure, such as age, body mass index (BMI), and a lack of prior chemotherapy treatment, but the small sample sizes prevented significant conclusions [12,17,18,19]. Therefore, more studies are needed to support these hypotheses.

Moreover, a clinical trial is currently underway to evaluate whether OFS combined with AI can substitute for chemotherapy in patients with pN1 disease or higher risk pN0 disease and an Oncotype DX recurrence score ≤ 25; thus, identifying women at risk of OFS failure has become increasingly important [20].

This study aims to determine the proportion of patients who experience OFS failure despite adequate treatment adherence, to identify factors associated with failure, to evaluate its impact on BC recurrence, and to examine whether failure occurs early (at treatment initiation) or later (after one year). Understanding these patterns may enable clinicians to individualize endocrine therapies and minimize unnecessary treatment-related side effects in selected patients.

## 2. Materials and Methods

**Study Design and Population** This monocentric retrospective cohort study included 208 premenopausal women diagnosed with HR + BC at the Centre des maladies du sein (CMS) of the CHU de Québec (Québec, Canada) between 2017 and 2021. Patients were excluded if they had previously undergone oophorectomy, were already menopausal at BC diagnosis, had metastatic BC, or received corticosteroids or fulvestrant while receiving endocrine therapy, due to potential interactions with estradiol level assays [21,22].

**Data Collection** Patients diagnosed between 2017 and 2021 were identified from the CMS breast cancer database, which provides detailed information on age, BMI, smoking status at diagnosis, tumor staging, histopathological characteristics, and treatments received. A total of 798 electronic medical records were screened, and only women who received OFS therapy were included. Demographic variables collected included menopausal status and presence of menstruation at diagnosis, age at menarche, and use of combined oral contraceptives. These variables were self-reported by patients using a standardized form, completed at the time of BC diagnosis. In cases of uncertainty regarding menopausal status, this was usually confirmed through hormonal blood tests ordered by the physician. Cancer recurrence, death, follow-up duration, details of endocrine therapy, including type, duration, cause of therapy cessation, and reported side effects were documented. Hormone levels, including E2, FSH, and LH, were measured to evaluate ovarian function. The presence of menstrual bleeding during treatment was documented by clinicians in the medical record.

**Outcomes** The primary outcome was OFS failure, defined as either E2 levels outside the thresholds established for menopause (biochemical failure), or clinical evidence of ovarian function recovery, defined by the return of menstruation (clinical failure). Hormone measurements were performed at different clinical laboratories, each with its own reference ranges for menopausal status. No standardized threshold exists to define estradiol levels in menopause, or in ovarian suppression failure [13]. In our center, competitive immunoassays are used, and the reference range for estradiol in menopausal women is <200 pmol/L. However, different immunoassays are used at regional hospitals, and their reference values therefore also differ (e.g., 110 pmol/L). To account for this, each patient’s hormone levels were interpreted according to the reference values of the lab where the analysis was performed. Secondary outcomes included identification of factors associated with OFS failure, timing of failure (before or after one year), BC recurrence, and survival. The exploratory objective concerned the assessment of side effects, that led to discontinuation of endocrine therapy.

**Statistical Analysis** Continuous variables are presented as the median, with interquartile range (IQR), while categorical variables are presented as frequencies and percentages. Associations between patients’ demographics, clinical characteristics, and treatment factors with OFS failure were assessed using Wilcoxon-Mann–Whitney tests for continuous variables, and Chi-square or Fisher’s exact tests for categorical variables. Time-to-event variables were calculated from the diagnosis date to the date of the first OFS failure. All statistical analyses were performed using SAS version 9.4, with a two-sided significance level set at *p* ≤ 0.05.

**Ethical Considerations** This project was made possible through an amendment to project 2018-3795 (Evaluation of diagnosis, treatments, follow-up, recurrences, and survival in patients with breast cancer at the CHU de Québec), which was previously approved by the CHU de Québec Research Ethics Committee on 18 June 2024. All collected data were stored on secure servers at the CHU de Québec.

## 3. Results

**Figure 1:**
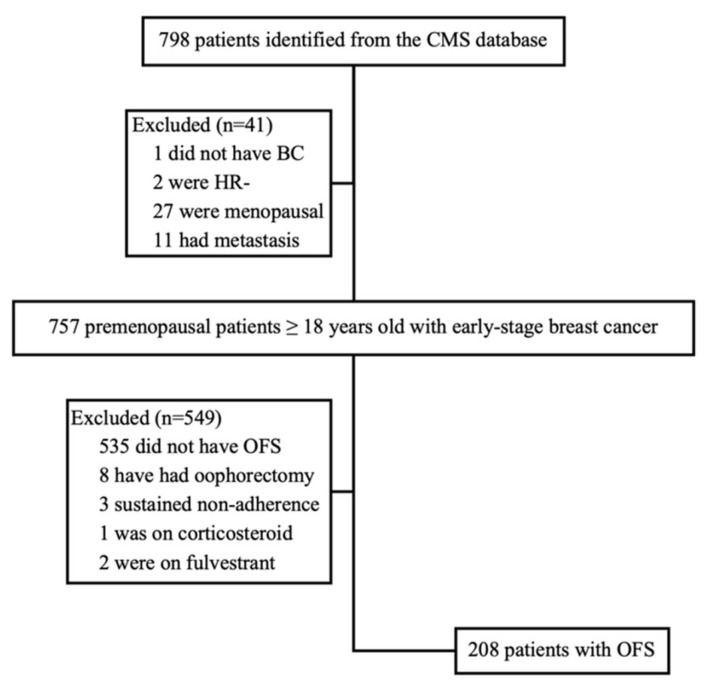
A study flow diagram illustrating the design of our cohort. Abbreviations: CMS, Centre des maladies du sein; BC, breast cancer; HR, hormone receptor; OFS, ovarian function suppression.

**Study population** A total of 798 patients’ medical records were reviewed. The most common reasons for exclusion were not receiving OFS therapy (67.0%) and being menopausal prior to the initiation of endocrine therapy (3.4%). After applying all inclusion and exclusion criteria, 208 patients were included in the final analysis (Figure 1), representing the main study cohort (Table 1).

All endocrine therapy regimens, including after OFS therapy cessation, are shown in Table 2.

The median age at diagnosis was 41.8 years (IQR 37.0–47.0), and the median BMI was 23.8 kg/m^2^ (IQR 21.4–27.3). Most patients had stage I BC (56.4%; stage information was missing for 6 patients) and received chemotherapy prior to OFS therapy (86.1%).

**Outcomes—OFS failure** Among the 208 patients, 17 patients (8.2%) experienced at least one episode of OFS failure during follow-up. Of the 17 patients, 3 experienced only biochemical failure (17.6%), 11 experienced only clinical failure (64.7%), and 3 exhibited both (17.6%) (Table 3).

A total of 21 distinct OFS failure events were documented across the 17 patients, reflecting the fact that some patients experienced more than one failure episode. Only one patient (patient K) experienced failure with two different OFS therapies.

Initial OFS therapy that subsequently led to treatment failure consisted of goserelin every month combined with an AI (anastrozole *n* = 12; letrozole *n* = 3). In one case, goserelin every 3 months was combined with tamoxifen due to injection-related symptoms, and in another, goserelin was used alone while awaiting AI initiation.

Several clinical strategies were used to manage cases of inadequate suppression, and clinicians may have used more than one strategy at a time (Table 4). The most common approach was gynecology referral (38.7%). Treatment modifications were also frequently implemented, including switching to goserelin plus tamoxifen (19.4%), tamoxifen alone (16.1%), and leuprolide (GnRHa) combined with an AI (12.9%). In some cases, temporary discontinuation of AI therapy with later resumption after resolution was reported (6.5%). Injection technique assessment was performed, and it was modified to be performed monthly by a nurse at a CLSC (6.5%). The outcomes of these different strategies are detailed in Table 4.

Endocrine therapies, and strategies details for each patient with OFS failure are also provided in Table S1 (Supplementary Materials).

The number of estradiol measurements available in the medical records was not the same for each patient, particularly those tested outside our institution, since access to the provincial health data repository was unavailable for research purposes. The number of measurements available for each patient was as follows: 0 for 33 patients, ≥5 for 106 patients, ≥10 for 56 patients, and ≥20 for 10 patients. Even when laboratory results were not directly accessible for our study, clinicians reported hormone values in the medical records, which enabled classification of OFS failure status.

**Timing of failure** The median time to OFS failure was 5.3 months (IQR 3.3–10). Among the 21 OFS failure episodes, 16 were early-failure (76.2%), occurring within the first year of treatment initiation, while 5 episodes (23.8%) were late-failure, occurring after at least 12 months of endocrine therapy.

**Factors associated with OFS failure** Bivariate analyses using Wilcoxon–Mann–Whitney tests showed no significant difference between patients with OFS failure and those with successful OFS regarding age (median 40.0 years vs. 42.0 years, *p* = 0.1488), BMI (median 25.8 kg/m^2^ vs. 23.7 kg/m^2^, *p* = 0.3128) or prior chemotherapy (70.6% vs. 87.4%, *p* = 0.0682) (Table 5).

However, the age at first pregnancy was significantly lower in the OFS failure group (median 23.79 years vs. 27.66 years, *p* = 0.0409), and active smoking at diagnosis was more frequent among patients with OFS failure (35.3% vs. 12.0%, *p* = 0.0180).

**Breast cancer recurrence** Over a median follow-up of 62.6 months (IQR 42.7–74.1), 25 patients (12.0%) experienced BC recurrence. No recurrences occurred in the OFS failure group. Among patients with recurrence in the successful OFS group, the most common site of recurrence was distant metastasis (*n* = 12, 48.0%), followed by local recurrence (*n* = 7, 28.0%). Other recurrence types are detailed in Table 6.

A total of 9 patients (4.3%) died during follow-up, none of whom were in the OFS failure group.

**Quality of life** Treatment discontinuations were assessed at the therapy level, with each attempted treatment counted separately. For example, a patient discontinuing two endocrine therapies contributed two discontinuation events. Reporting discontinuations per therapy rather than per patient allows for a clearer assessment of tolerability independent of the number of therapies tried by individual patients. Across the cohort, 348 endocrine therapies were recorded among 208 patients. Treatment-related side effects were frequently reported (Table 7), with 80 discontinuation events documented, corresponding to 23.0% of therapies being stopped by patients due to side effects.

The most common reasons for discontinuation were arthralgia (26 events, 33.0%), mood disturbances (9 events, 11.3%), fatigue, and hot flushes (8 events each, 10.0%).

## 4. Discussion

In this monocentric retrospective cohort study of 208 premenopausal patients undergoing endocrine therapy for HR + BC, 17 patients (8.2%, *n* = 208) experienced at least one episode of OFS failure during follow-up, defined as E2 levels outside the menopausal values or return of menstruation. This incidence is slightly lower than that reported in previous studies, including Lin et al. (5–50% depending on the E2 threshold, *n* = 17 studies included), Burns et al. (24%, *n* = 46), SOFT-EST (17%, *n* = 116), and Patel et al. (20%, *n* = 85) [16,17,23,24]. These differences highlight the heterogeneity in OFS failure definition and monitoring strategies.

Several factors may explain the lower OFS failure rate observed in our study. In Québec, since GnRHas are typically administered by a trained nurse every 4 weeks in a local community services center (CLSC), it ensures standardized injection technique, which could potentially reduce the risk of suboptimal drug delivery and OFS failure. Two patients in our study who initially experienced OFS failure were self-administering their injections, and OFS failure was corrected once administration was performed by a nurse. This observation suggests that self-injection, particularly given the large needle size required for goserelin, may necessitate thorough patient education.

Differences in menopausal status assessment may also have contributed to our lower failure rate. In our study, menopausal status was determined at BC diagnosis, prior to chemotherapy, whereas SOFT-EST classified menopausal status after chemotherapy [23]. We do not systematically reassess menopausal status with hormonal dosage following chemotherapy, before initiating endocrine therapy. This suggests that a proportion of patients in our cohort may not have been at risk of OFS failure, because ovarian function had already ceased due to chemotherapy.

The timing of OFS failure is consistent with prior research. In our study, most failures occurred before one year (76.2%), and the median time to OFS failure was 5.3 months. This result goes along with a previous study suggesting that incomplete OFS rate decreased with the extension of treatment time [16]. Some patients in our cohort experienced OFS failure across multiple endocrine therapies, suggesting that switching therapies does not uniformly guarantee adequate OFS [25].

Despite the occurrence of OFS failure, no BC recurrences or deaths were observed among patients with documented failure during the follow-up period. This finding must be interpreted cautiously, given the small sample size and limited number of events in this subgroup. Recurrences and mortality were observed exclusively among patients with sustained OFS, likely reflecting the overall distribution of patients rather than a protective effect of OFS failure.

Although prior studies have identified younger age, higher BMI, and the absence of prior chemotherapy as potential risk factors for OFS failure, we did not observe statistically significant associations for these variables in our study [15,16,24]. This is likely explained by the small number of failures in our cohort, which limited statistical power. Our study does not allow for definitive confirmation or refutation of previously reported risk factors.

We found that patients experiencing OFS failure had a significantly younger age at first pregnancy. To our knowledge, there is currently no established evidence in the literature supporting a causal relationship between age at first pregnancy and OFS failure. Existing studies primarily describe early age at first pregnancy as a protective factor against the development of BC, rather than as a determinant of endocrine treatment response [26,27]. Given the exploratory nature of this finding, this result should be interpreted with caution and may represent a chance association. Additional studies with larger sample sizes and dedicated endocrine evaluations are required to determine whether age at first pregnancy has any true influence on OFS therapy efficacy.

We also found that patients experiencing OFS failure had higher rates of active smoking at the time of BC diagnosis, an unexpected finding given the association between smoking and diminished ovarian reserve [28]. Smoking may influence estrogen metabolism through cytochrome P450 induction and modulation of aromatase activity [28], although its impact on GnRHa efficacy remains unclear [26,29,30]. Evidence on smoking and endocrine therapy outcomes is limited, with some studies suggesting reduced efficacy of AIs in smokers but no clear effect with tamoxifen [27]. Evidence from postmenopausal populations suggests a potential association between smoking and estrogen levels, whereas data in premenopausal women remain limited, warranting further investigation [31,32,33].

A total of 76 events of discontinuation out of 318 endocrine treatments were recorded. This number could be overestimated, given that adherence to treatment is probably underreported. This raises concerns as to whether OFS plus AI can replace chemotherapy, as being currently studied in the OFSET trial [20]. These results support the need for individualized monitoring strategies and reinforce the importance of considering both biochemical markers and clinical symptoms when assessing OFS efficacy.

**Strengths and limitations** This study benefits from a long follow-up period exceeding five years (62.6 months). Detailed documentation of treatment discontinuations enabled an evaluation of the tolerability of endocrine therapy.

Our study was limited by a relatively small number of OFS failure events, which limits statistical power and precludes robust multivariable analyses. Although a standardized data collection tool was used to harmonize chart review across investigators, the retrospective and observational design relied on the completeness and accuracy of medical records, resulting in some missing data despite the use of standardized clinical forms in routine practice. There was no formal patient-reported outcome assessment, so side effects were collected from healthcare professional notes. Treatment adherence was difficult to assess, as GnRHa injections were administered outside our institution, but patients were followed regularly at our center, allowing clinicians to address adherence during scheduled visits.

## 5. Conclusions

Tamoxifen or AIs paired with GnRHas have been shown to reduce the risk of recurrence, but these treatments do not consistently achieve complete OFS in all patients. In our study, 8.2% of premenopausal women experienced OFS failure during follow-up. This study provides real-world evidence that adds to the growing literature on OFS failure, supporting observations from prior small studies and underscoring the need for larger prospective investigations to better define its incidence, risk factors, and clinical implications.

## Figures and Tables

**Table 1:** Baseline characteristics (*n* = 208).

Characteristics	
Age at diagnosis, years (median, IQR)	41.8 (37.0–47.0)
BMI, kg/m^2^ (median, IQR)	23.8 (21.4–27.3)
Menarche, years (median, IQR)	13.0 (12.0–13.0)
Missing	3
Age at first pregnancy (median, IQR)	27.6 (24.6–30.3)
No pregnancy	32
Missing	18
Smoking history	*n* (%)
Active smoker	29 (13.9)
Former smoker	63 (30.0)
Never	116 (55.8)
Contraception	*n* (%)
Former combined oral contraception	183 (92.0)
Never	16 (8.0)
Missing	9
Number of years (median, IQR)	12 (7.0–18.0)
History of BC (before the actual BC being treated with OFS)	*n* (%)
Previous BC	8 (3.9)
Never	198 (96.1)
Missing	2
TNM classification	*n* (%)
0 *	1 (0.5)
IA	61 (29.3)
IB	53 (25.5)
IIA	41 (19.7)
IIB	24 (11.5)
IIIA	13 (6.4)
IIIB	6 (3.0)
IIIC	3 (1.5)
Missing	6
HR expression	*n* (%)
Estrogen +	208 (100)
Estrogen −	0 (0)
Progesterone +	184 (88.5)
Progesterone −	24 (11.5)
HER2 status	*n* (%)
HER2+	42 (20.6)
HER2−	162 (79.4)
Missing	4
Chemotherapy	*n* (%)
Yes	179 (86.1)
No	29 (13.9)
Radiotherapy	*n* (%)
Yes	181 (87.0)
No	27 (13.0)

* *A patient with extensive carcinoma in situ was put on OFS after a recurrence while on tamoxifen.* Abbreviations: IQR, interquartile range; BMI, body mass index; BC, breast cancer; HR, hormone receptor; HER2, human epidermal growth factor receptor 2.

**Table 2:** Types of endocrine therapies regimens, including after OFS cessation (*n* = 426).

Types	OFS Failure Number of Patients = 17 Endocrine Therapies Attempt = 42	Succeed OFS Number of Patients = 191 Endocrine Therapies Attempt = 384
Goserelin * + AIs; *n* (%)	16 (38.1%)	207 (53.9%)
Goserelin * + Tamoxifen; *n* (%)	8 (19.0%)	32 (8.3%)
Leuprolide + AIs; *n* (%)	5 (11.9%)	0 (0%)
Tamoxifen; *n* (%)	8 (19.0%)	66 (17.2%)
AIs; *n* (%)	3 (7.1%)	62 (16.1%)
Others **; *n* (%)	2 (4.8%)	17 (4.4%)

* *Goserelin is routinely administered at a dose of 3.6 mg every 4 weeks, which represents the standard of care and is expected to have been prescribed for all patients in our cohort.* ** *Including combinations with cyclin D1/CDK4 and CDK6 inhibitors.* Abbreviations: AIs, aromatase inhibitors.

**Table 3:** Duration in months of OFS therapy prior to OFS failure (*n* = 17).

Patients	Clinical Failure	Biochemical Failure
A	**5.8**	**5.2**
B	41.7	No biochemical failure
C	No clinical failure	50.1
D	**3.3**	**2.6**
E	**1.7**; **6.7**; 54.9	No biochemical failure *
F	**11.1**	No biochemical failure
G	**6.2**	No biochemical failure
H	No clinical failure	**4**
I	**3.8**	No biochemical failure
J	12.2	No biochemical failure *
K	**3**; **5**	**4.3**
L	No clinical failure	**1.6**
M	29.2	No biochemical failure
N	**8.9**	No biochemical failure
O	**3.4**	No biochemical failure
P	**3.1**; **5.4**	No biochemical failure *
Q	**5.4**	No biochemical failure

*The number in bold represents early failure (<1 year).* * *According to the medical records (laboratory results not directly accessible).* Abbreviations: OFS, ovarian function suppression. The number in bold represents early failure (<1 year).

**Table 4:** Strategies employed by clinicians (*n* = 31) and outcomes for patients with OFS failure.

Strategies and Outcomes	
**Temporary discontinuation of the AI, with same treatment resumed after resolution**; *n* (%) Adequate suppression achieved (*n* = 2)	2 (6.5%)
**Switch to Leuprolide (GNRHa) and AI**; *n* (%) Recurrence of bleeding 2 months later (*n* = 1) Adequate suppression achieved (*n* = 3) Switch to Goserelin and Tamoxifen; *n* (%) Recurrence of bleeding several months later (*n* = 1)	4 (12.9%)
**Switched to Tamoxifen only due to persistent failure** (*n* = 1) Estradiol levels at the upper limit of normal (*n* = 1) Repeated gynecological investigations performed (*n* = 2) Adequate suppression achieved (*n* = 1)	6 (19.4%)
**Switch to Tamoxifen alone**; *n* (%) No estradiol level follow-up	5 (16.1%)
**Injection technique assessment**; *n* (%) Adequate suppression achieved (*n* = 2)	2 (6.5%)
**Gynecology referral**; *n* (%) Hysterectomy and bilateral salpingo-oophorectomy (*n* = 1) Endometrial thickening (*n* = 2) Negative investigations (*n* = 9)	12 (38.7%)

*In some cases, more than one strategy may have been employed for a single failure.* Abbreviations: OFS, ovarian function suppression; Ais, aromatase inhibitors; GnRHas, Gonadotropin-releasing hormone agonists.

**Table 5:** Comparison of characteristics of patients with OFS failure and successful OFS (*n* = 208).

	Patients with OFS Failure (*n* = 17)	Patients with Successful OFS (*n* = 191)	*p* Value
Age at diagnosis, years (median, IQR)	40.0 (32.0–42.0)	42.0 (37.0–47.0)	0.15
BMI, kg/m^2^ (median, IQR)	25.8 (22.1–27.7)	23.7 (21.3–27.1)	0.31
Menarche, years (median, IQR)	12.0 (11.0–13.0)	13.0 (12.0–13.5)	0.24
Age at first pregnancy (median, IQR)	23.8 (21.3–29.2)	27.7 (24.9–30.7)	0.04
Smoking history; *n* (%)			0.02
Active smoker	6 (35.3)	23 (12.0)	
Former smoker	2 (11.8)	61 (31.9)	
Never smoked	9 (52.9)	107 (56.0)	
Contraception; *n* (%)			1.00
Active/former contraception	16 (94.1)	170 (91.9)	
Never	1 (5.9)	15 (8.1)	
Number of years (median, IQR)	14 (7.0–15.0)	12 (7.0–18.0)	
History of BC; *n* (%)			0.63
Previous BC	0 (0.0)	8 (4.2)	
Never	17 (100.0)	181 (95.8)	
TNM classification; *n* (%)			0.33
0	0 (0.0)	1 (1.5)	
IA	5 (29.4)	56 (29.3)	
IB	4 (23.5)	49 (25.7)	
IIA	5 (29.4)	36 (18.8)	
IIB	1 (5.9)	23 (12.0)	
IIIA	0 (0.0)	13 (6.8)	
IIIB	2 (11.8)	4 (2.1)	
IIIC	0 (0.0)	3 (1.6)	
HR expression; *n* (%)			1.00
Estrogen +	17 (100)	191 (100)	
Estrogen −	0 (0)	0 (0)	
Progesterone +	15 (88.2)	169 (88.5)	
Progesterone −	2 (11.8)	22 (11.5)	
HER2 status; *n* (%)			1.00
HER2+	3 (17.6)	39 (20.9)	
HER2−	14 (82.4)	148 (79.1)	
Chemotherapy; *n* (%)			0.07
Yes	12 (70.6)	167 (87.4)	
No	5 (29.4)	24 (12.6)	
Radiotherapy, *n* (%)			0.25
Yes	13 (76.5)	168 (88.0)	
No	4 (23.5)	23 (12.0)	

Abbreviations: OFS, ovarian function suppression; IQR, interquartile range; BC, breast cancer; HR, hormone receptor; HER2, human epidermal growth factor receptor 2.

**Table 6:** Types of recurrence (*n* = 25).

Types	Recurrence in Patients with OFS Failure	Recurrence in Patients Without OFS Failure
Local; *n* (%)	0 (0%)	8 (32.0%)
Regional; *n* (%)	0 (0%)	2 (8.0%)
Distant metastasis; *n* (%)	0 (0%)	12 (48.0%)
Contralateral BC; *n* (%)	0 (0%)	3 (12.0%)

Abbreviations: BC, breast cancer.

**Table 7:** Number of endocrine therapy discontinuation events and associated side effects (*n* = 348).

	OFS Failure Number of Patients = 17 Endocrine Therapy Attempts = 31	Succeed OFS Number of Patients = 191 Endocrine Therapy Attempts = 317
Number of therapies ceased due to side effects	4	76
Reported side effects	Headache: 3 Dysthymia: 1	Arthralgia: 26 Mood disturbance: 9 Fatigue: 8 Hot flushes: 8 Dysthymia: 7 Pain: 3 Nausea: 3 Decreased libido: 2 Others: 10

*Note: Discontinuations are counted per therapy attempt. A single patient may contribute more than one event if multiple regimens were discontinued.* Abbreviations: OFS, ovarian function suppression.

## Data Availability

Anonymized data can be made available upon reasonable request to the corresponding author.

## Supplementary Materials

The following supporting information can be downloaded at: https://www.mdpi.com/article/10.3390/cancers18081296/s1, Table S1: Types of therapies and clinician strategies for patients with ovarian suppression failure.

## Abbreviations

The following abbreviations are used in this manuscript:

AIs	Aromatase inhibitors
BC	Breast cancer
BMI	Body mass index
CLSC	Local community services center
CMS	Centre des maladies du sein
DFS	Disease-free survival
E2	Estradiol
FSH	Follicle-stimulating hormone
GnRHas	Gonadotropin-releasing hormone agonists
HER2	Human epidermal growth factor receptor 2
HR	Hormone receptor
HR+	Hormone receptor-positive
IQR	Interquartile range
LH	Luteinizing hormone
OFS	Ovarian function suppression
