# Supplementary material for: Evaluation of Ovarian Function Suppression Failure in Premenopausal Women with Early-Stage Breast Cancer
Source: Cancers (Basel). 2026 Apr 20;18(8):1296. doi: 10.3390/cancers18081296 (PMC13115181; doi:10.3390/cancers18081296)
Supplement: Supplementary file 1 [file cancers-18-01296-s001.zip › cancers-4241672-supplementary.pdf]

Supplementary Material

Table S1. Types of therapies and clinician strategies for patients with ovarian suppression failure.

Patients	Failures
A	**Failure #1**<br>**Type**: Clinical and biochemical failure at 5.2 and 5.8 months.<br>**Therapy**: Goserelin + Anastrozole.<br>**Strategy**: Temporary discontinuation of the AI; patient from an outside facility and therefore injection procedure unknown.<br>**Result**: No new bleeding and normalization of hormone levels. AI restarted 3 months later.
B	**Failure #1**<br>**Type**: Clinical failure at 41.7 months with normal hormonal levels.<br>**Therapy**: Goserelin + Anastrozole.<br>**Strategy**: Temporary discontinuation of the AI; investigated for endometrial thickening and a non-specific coagulation disorder.<br>**Result**: AI restarted. No recurrence of bleeding thereafter and normal hormonal levels.
C	**Failure #1**<br>**Type:** Biochemical failure at 50.1 months without clinical failure.<br>**Therapy**: Goserelin + Anastrozole.<br>**Strategy:** Temporary discontinuation of anastrozole, followed by a switch to Tamoxifen alone. Referral to gynecology.<br>**Result:** No estradiol level follow-up.
D	**Failure #1**<br>**Type:** Clinical and biochemical failure at 2.6 and 3.3 months.<br>**Therapy**: Goserelin + Anastrozole.<br>**Strategy:** Temporary discontinuation of anastrozole, followed by a switch to Tamoxifen alone.<br>**Result:** No new bleeding and no estradiol level follow-up.
E	**Failure #1**<br>**Type**: Clinical failure at 1.7 months without biochemical failure according to the medical records (laboratory results not directly accessible).<br>**Therapy:** Goserelin alone (awaiting initiation of IA therapy).<br>**Strategy:** Goserelin + Tamoxifen.<br>**Result:** Recurrence of bleeding 5 months later (failure 2).<br><br>**Failure #2**<br>**Type:** Clinical failure at 6.7 months without biochemical failure according to the medical records (laboratory results not directly accessible).<br>**Therapy:** Goserelin + Tamoxifen.<br>**Strategy:** Adherence confirmed, and gynecological investigations negative.<br>**Result:** No recurrence of bleeding for 4 years.<br><br>**Failure #3**<br>**Type:** Clinical failure at 54.9 months without biochemical failure according to the medical records (laboratory results not directly accessible).<br>**Therapy:** Goserelin + Tamoxifen.<br>**Strategy:** No change. Gynecological investigations negative again.<br>**Result:** No recurrence of bleeding to date.
F	**Failure #1**<br>**Type**: Clinical failure at 11.1 months with normal hormonal levels.<br>**Therapy:** Goserelin + Anastrozole.<br>**Strategy:** Goserelin + Tamoxifen.<br>**Result:** No recurrence of bleeding thereafter. Estradiol levels at the upper limit of normal (e.g., 167 pmol/L; reference range < 200 pmol/L).
G	**Failure #1**<br>**Type**: Clinical failure at 6.2 months with normal hormonal levels.<br>**Therapy:** Goserelin + Anastrozole.

	**Strategy:** Leuprolide (GNRHa) + Anastrozole. Referral to gynecology.<br>**Result:** Adequate suppression achieved.
H	**Failure #1**<br>**Type:** Biochemical failure at 4 months without clinical failure.<br>**Therapy:** Goserelin + Anastrozole.<br>**Strategy:** Initial change to Goserelin + Tamoxifen, then switched to tamoxifen for four months due to persistent failure.<br>**Result:** No estradiol level follow-up.
I	**Failure #1**<br>**Type**: Clinical failure at 3.8 months with normal hormonal levels.<br>**Therapy:** Goserelin + Anastrozole.<br>**Strategy:** Gynecologic evaluation.<br>**Result:** No recurrence of bleeding thereafter with normal hormonal levels.
J	**Failure #1**<br>**Type**: Clinical failure at 12.2 months without biochemical failure according to the medical records (laboratory results not directly accessible).<br>**Therapy:** Goserelin every 3 months + Tamoxifen. Exceptional situation in Quebec; the patient was symptomatic from the injections.<br>**Strategy:** Goserelin monthly + Tamoxifen and referral to gynecology.<br>**Result:** No recurrence of bleeding thereafter.
K	**Failure #1**<br>**Type:** Clinical failure at 3 months with biochemical failure at 4.3 months.<br>**Therapy:** Goserelin + Letrozole.<br>**Strategy:** Change to Leuprolide + Letrozole.<br>**Result:** Recurrence of bleeding 2 months later.<br><br>**Failure #2**<br>**Type**: Clinical failure at 5 months.<br>**Therapy:** Leuprolide + Letrozole.<br>**Strategy**: Tamoxifen and referral to gynecology.<br>**Result**: No bleeding monitoring or estradiol level follow-up.
L	**Failure #1**<br>**Type:** Biochemical failure at 1.6 months without clinical failure.<br>**Therapy:** Goserelin + Anastrozole.<br>**Strategy:** Young patient living outside the city who was self-administering her injections; subsequently switched to administration by a nurse at a local clinic (CLSC).<br>**Result:** Adequate suppression achieved.
M	**Failure #1**<br>**Type:** Clinical failure at 29.2 months with normal hormonal levels.<br>**Therapy:** Goserelin + Anastrozole.<br>**Strategy:** Change to Goserelin + Tamoxifen and referral to gynecology.<br>**Result:** No recurrence of bleeding thereafter. Laboratory results not directly accessible (reported normal in the medical records).
N	**Failure #1**<br>**Type**: Clinical failure at 8.9 months with normal hormonal levels.<br>**Therapy:** Goserelin + Tamoxifen initially for tolerance and switched to Goserelin + Letrozole.<br>**Strategy:** Given the recent change (2 months) in therapy, no further changes were made. Classified as treatment failure since the patient was already receiving goserelin. Referral to gynecology.<br>**Result:** No recurrence of bleeding thereafter with normal hormonal levels.
O	**Failure #1**<br>**Type**: Clinical failure at 3.4 months with normal hormonal levels.<br>**Therapy:** Goserelin + Anastrozole.<br>**Strategy:** Change to Tamoxifen and underwent hysterectomy for a polyp and bilateral salpingo-oophorectomy.<br>**Result:** No recurrence of bleeding thereafter.
P	**Failures #1-2**

	**Type**: Clinical failure at 3.1 and 5.4 months without biochemical failure according to the medical records (laboratory results not directly accessible).<br>**Therapy:** Goserelin + Anastrozole.<br>**Strategy:** Leuprolide + Anastrozole after the second clinical failure and gynecological investigations negative.<br>**Result:** No recurrence of bleeding thereafter. Laboratory results not directly accessible.
Q	**Failure #1**<br>**Type:** Clinical failure at 5.4 months with normal hormonal levels.<br>**Therapy:** Goserelin + Letrozole.<br>**Strategy:** Leuprolide + Letrozole and referral to gynecology.<br>**Result:** No recurrence of bleeding thereafter with normal hormonal levels.

Abbreviations: OFS, ovarian function suppression; AI, aromatase inhibitor; GnRHa, Gonadotropin-releasing hormone agonist.
